# 

**Published:** 1842-01-01

**Authors:** 


### MEDICO-CHIRURGICAL

### REVIEW,

AND

### JOURNAL

OF

### PRACTICAL MEDICINE.

*(NEW SERIES.)*

VOLUME THIRTY-SIX,

[1st of OCTOBER, 1841, to 31st of MARCH,]

1842.

VOL. XVI. of DECENNIAL SERIES.

EDITED

BY JAMES JOHNSON, M.D.

PHYSICIAN EXTRAORDINARY TO THE LATE KING,

AND

HENRY JAMES JOHNSON, ESQ.

LECTURER ON ANATOMY AT THE SCHOOL OF ST. GEORGE'S HOSPITAL IN KINNERTON STREET.

Surgeon Genl's Office LIBRARY. Washington, D.C. 57476

LONDON:

PUBLISHED BY S. HIGHLEY, 32, FLEET STREET,

*Re-printed in New York, by Mr. Wood.*

1842.

VU1
ME 75 T

PRINTED BY F. HAYDEN,
Little College Street, Westminster.

### INDEX.

A.

Abdominal effusion, Hughes' case of 233
Abdominal effusion, Dr. Rees on .... 233
Abscess of the iliac region, Velpeau on 549
Abscesses, Sir B. Brodie on ........ 557
Academy of medicine, memoirs of the 369
Acute aortitis, Chevers on.......... 234
Africa, coast of .................. 359
Albuminous ascites with hydatids .. 289
Albuminuria ..................... 452
Amaurosis, section of muscles in.... 209
Amaurosis, hysterical, Hocken on.... 491
Amenorrhœa ..................... 380
Amputation, Potter on the results of 34
Amputation of the fore-arm........ 173
Amputation of the thigh .......... 174
Amputation of the leg ........... 175
Amputation during traumatic gangrene 552
Amputations, double-flap and circular 172
[illegible]næmia ..................... 519
[illegible]naphrodisia, influence of cerebellum 280
Anatomy of the lungs, Addison on the 32
Anatomy, cyclopædia of ... ...... 140
[illegible]natomy, studentships in.......... 299
Anatomy of the brain, Foville on.... 401
Andral on alterations of the blood . 181
Andral on the changes in blood...... 518
Aneurism of sup. mesenteric ...... 45
Aneurysm, operations for.......... 243
Aneurysm, popliteal, ligature in .... 243
Aneurysm of the carotid .......... 375
Aneurysm, axillary, case of ........ 548
Aneurysm of the aorta ............ 573
Animal magnetism, prohibition of, by the Vatican .................. 213
Animal tissues, Toynbee on . ...... 426
Animals, cerebrum and cerebellum in 169
Animals, spinal marrow in ........ 170
Annan's clinical report ............ 255
Annual report of the registrar-general 305
Anthelmintics in Abyssinia ........ 402
Anus, artificial, formation of an,.... 204
Aortitis, causes of ................ 237
Aortitis, symptoms of ............ 237
Apoplexy ...................... 19
Arm, amputation of the .......... 172
Arsenic, Taylor on poisoning with .. 231
Arsenic, ulceration of stomach from 232
Arsenic, Orfila on poisoning by .... 371
Arsenic, detection of .............. 371
Arseniuretted hydrogen, poisoning by 569
Art of percussion ............... 563
Arteria carotidea, ligature of the .... 244
Articular cartilage, structure of...... 428
Articular cartilage, development of .. 429
Articular cartilage in the adult...... 431
Articular rheumatism ............ 414
Artificial anus .................. 204
Ascites and ovarian dropsy, diagnosis of 558
Auscultation, Fournet on .......... 455
Auscultatory signs of pregnancy .... 197
Austria, state of surgery in . ...... 262
Austria, ophthalmology in.......... 262
Axillary aneurysm, case of ........ 548
Axis, dislocation of the............ 109

B.

Babington on chorea.............. 251
Baltimore hospital, report from .... 255
Barlow on diseases in early youth .. 225
Baron Larrey's career ............ 202
Barth, a teacher of ophthalmology .. 262
Base of the encephalon, special anatomy of the ........ ........ 23
Bennett on the microscope ........ 165
Bicipital tendon, Sodon on displacement of ...................... 42
Bingham on delusions of the insane.. 423
Birkbeck, memoir of .............. 494
Births, deaths and marriages ...... 305
Bladder, Civiale on neuralgia of the.. 208
Bladder, Willis on stone in the...... 467
Bladder, injections into the ........ 473
Blindness, section of muscles in .... 211
Blindness cured by operation ...... 274
Blood, Andral on alterations of the.. 181
Blood corpuscle, Rees on the........ 248
Blood corpuscle, Lane on the ...... 248
Blood, Andral on the changes in .... 518
Blood in fevers ................. 520
Boismont on menstruation ........ 377
Boyd's case of deficiency of the uterus 41
Brady on auscultation ............ 455
Brain, concussion of the .......... 246
Brain, Foville on the anatomy of the 401
Brandy and salt ................. 163
Bricheteau on epidemics in France .. 369
Brodie, Sir B. on abscesses ....... 557
Bronchi, diseases of the . ........ 464
Bronchitis, case of................ 227
Bronchitis, acute and chronic ... .. 464
Broussais, inauguration of the statue of 510
Burns, treacle and water for........ 551
Buxton ........................ 78
Buxton, effects of the waters of .... 82

C.

Cambray wells .................. 97
Campbell on consumption ........ 129
Cancer of spinal column, Hawkins on 10
Cancer of the womb, Montgomery on 568
Cancer of stomach, Dr. Watson on.. 551
Cape of Good Hope .............. 359
Capsular rheumatism.............. 414

Capsular rheumatism, treatment of.. 418
Carditis and pericarditis .......... 409
Carlisle, medical charities of ...... 277
Carotid artery, ligature of the ...... 244
Carotid, ligature of the............ 375
Carotid, aneurysm of the.......... 375
Carpenter, Dr. letter from.......... 293
Case of slow pulse................ 16
Causes of death in England........ 308
Centralization, effects of .......... 177
Cerebellum, influence of, on anaphrodisia.......................... 280
Cerebellum, Jackson on disease of the 570
Cerebrum and cerebellum in animals 169
Cervix uteri, fracture of the........ 3
Cervix uteri, changes in the ........ 198
Cheltenham ...................... 93
Cheltenham, old well at .......... 94
Cheltenham, analyses of waters at .. 95
Chemists, medical reform for ...... 162
Chest, fistulous opening in the...... 217
Chevers on acute aortitis .......... 234
China, midwifery in .............. 565
Chlorosis.......................... 381
Chorea, Babington on ............ 250
Chorea, definition of .............. 252
Chorea and cardiac affections, connexion between ................ 253
Chorea, treatment of .............. 253
Chorea, use of tonics in .......... 254
Circular amputation .............. 172
Circular and flap operations, Mr. Fergusson on .................... 560
Circumcision at Constantine........ 532
Civiale on neuralgia of the bladder .. 208
Civiale, sketch of ................ 475
Claridge on hydropathy.......... .... 362
Clark on the purification of water .. 275
Clinical report, Annan's .......... 255
Clinical report, Davidson's ........ 258
Clinique, Jäger's ................ 263
Clinique of Rosas ................ 265
Closure of the os uteri ............ 573
Clutterbuck's memoir of Dr. Birkbeck 494
Coast of Africa .................. 359
Colica pictonum, Dr. Wilson on .... 40
Colon, scirrhus of the ............ 266
Commissioners of lunacy, report of.. 554
Comparative anatomy, Swan's...... 168
Concussion of the brain .......... 246
Congenital tumors of pelvis, Stanley on 48
Congenital dislocations ............ 109
Consumption, causes of .......... 129
Consumption, treatment of ........ 130
Consumption, pathology of ........ 130
Consumption, predisposing causes of 542
Cooper on operations.............. 242
Cooper on dislocations ............ 496
Cornea, upper section of the........ 263
Cornea, structure of the .......... 435
Cornea, nourishment of the ........ 436
Country, diseases of the .......... 319
Crommelinck, letter from.......... 303
Crook on medical reform .......... 162
Cruveilhier on the sounds of the heart 193
Crystalline lens, anatomy of the .... 437
Curvatures of the spine .......... 279
Cutaneous affections, Davidson on .. 268
Cyclopædia of anatomy .......... 140
Cyclopædia of practical surgery .... 149
Cystectasy ........................ 489
Cysts in the kidneys .............. 220

D.

Davidson's clinical report .......... 258
Davidson on cutaneous affections.... 268
Death, causes of, in England ........ 308
Diabetes mellitus, treatment of...... 534
Diabetes, Bouchardat on .......... 534
Diagnosis of tumors .............. 266
Dick on derangements of the organs of digestion........................ 64
Dickson's remarkable cases ........ 289
Disarticulation of the humerus...... 376
Diseases of the kidneys, Rayer on.... 51
Diseases in early youth, Barlow on .. 225
Diseases of towns and country...... 319
Dislocation of the axis ............ 109
Dislocation of the humerus, reduction 403
Dislocations, spontaneous, Stanley on 26
Dislocations, congenital............ 109
Dislocations, Sir A. Cooper on ...... 496
Diuretics in phthisis, &c. .......... 559
Donellan on erysipelas ............ 157
Double-flap and circular amputation.. 172
Dubois on the auscultatory signs of pregnancy ...................... 197
Dundee lunatic asylum, report ...... 545
Dysentery ........................ 370
Dysmenorrhœa .................... 381
Dyspeptic's melancholy tale ........ 66
Dyspeptic phthisis.................. 66

E.

Ear, Toynbee on diseases of the.. .. 42

Early youth, Barlow on diseases in .. 225

Effects of centralization............ 177

Embryo, venous system in the ...... 390

Encephalocele, Wells on .......... 150

Encephalocele, causes of .......... 150

Encephalocele, seats of ............ 151

Encephalocele, diagnosis of ........ 151

Encephalocele, treatment of........ 153

Endosteitis, Walshe on ............ 154

England, Granville on the spas of.... 68

England, toxicology in ............ 231

England, pharmacy in ............ 285

England, mortality in.............. 305

England, causes of death in ........ 308

Entozoa, Gulliver on the .......... 2

Epidemic diseases, Parkin on........ 104

Epidemics in France .............. 369

Epidermoid tissues................ 440

Epilepsy .......................... 20

Epiphysal cartilages .............. 429

Epistaxis, Wells on.................. 156

Erysipelas, Donellan on............ 157

Erysipelas, influence of seasons on .. 157

Erysipelas, contagiousness of ...... 157

Erysipelas, disappearance of........ 158

Erysipelas, Blandin on ............ 158

Erysipelas, mercurial ointment for .. 159

Erysipelas, treatment of............ 160

Excision of the elbow joint ........ 245

Expiratory murmur ................ 457

Exposure to cold, effects of ........ 24

Extra-vascular tissues ............ 438

F.

Farcy, Rayer on.................... 160

Farcy, transmission of ............ 532

Fascial nerves, palsy of the ........ 199

Fees to general practitioners........ 285

Fever, Hudson on .................. 273

Fevers, on the blood in.............. 520

Fibro-cartilage .................... 433

Fibro-cartilage, nourishment of .... 434

Finances of the College of Surgeons 302

Fish-liver oil, properties of ........ 215

Fistula............................ 160

Fistula, Hingeston on ............ 249

Fistulous opening in the chest...... 217

Foreign bodies in the kidneys ...... 223

Formation of an artificial anus...... 204

Fournet on auscultation............ 455

Foville on the anatomy of the brain.. 401

Fracture of the cervix uteri, Jones on 3

Fracture of the patella, King on .... 247

Fractures, Sir A Cooper on........ 496

France, suicide in ................ 198

France, pharmacy in .............. 283

France, epidemics in .............. 369

Franz's operation for blindness...... 274

French physic, Wunderlich on...... 177

Fungoid disease of the lungs........ 239

Fungoid disease of the thigh........ 241

G.

Galen, Dubois' account of.......... 521

Galen, important discoveries of...... 524

Galen's case of hepatitis .......... 527

Gangrene of the lungs ............ 256

Gangrenous affections in the puerperal state.............................. 185

General practitioners, fees to ...... 285

Gestation of the marsupialia........ 141

Glanders, cases of ................ 188

Glanders, transmission of.......... 532

Glasgow Royal Infirmary .......... 258

Gonorrhœa, nitrate of silver in...... 281

Good Hope, Cape of, report from.... 359

Gouty concretions, Ure on.......... 6

Gouty nephritis .................. 56

Grace, Val de, hospital of the ...... 510

Granular disease of the kidneys .... 447

Granular kidney, nature of ........ 448

Granular kidney, causes of.......... 457

Granville's Spas of England........ 68

Greek medical authors ............ 521

Gregory on vaccination...... ...... 4

Guerin on subcutaneous surgery .... 109

Gulliver on the entozoa............ 2

Guy's Hospital Reports............ 225

H.

Hæmaturia in the Isle of France .... 62

Hæmaturia, endemic .............. 62

Hall on the nervous system ........ 18

Hall on the use of setons .......... 260

Hanging, Orfila on................ 384

Harrogate ........................ 70

Hawkins on cancer of spinal column 10

Hayden's physiology for the public .. 161

Health of the navy................ 358

Heart, Cruveilhier on the sounds of.. 193

Heart, congenital mal-formation of the 193

Heart, movements of the .......... 193

Heart, case of partial hypertrophy of 276

Hemiplegia ...................... 19

Hemiplegia, case of.............. 255

Hepatic abscess, cases of .... .... 290

Hepatitis, Galen's case of .......... 527

Hernia, simulation of.............. 267

Hiccup cured with quinine ........ 192

Hingeston on fistula .............. 249

Hip, dislocation of the ............ 271

Hip-joint, dislocations of the........ 26

Hip-joint, voluntary dislocation of the 31

History of the venous system ...... 387

Hocken on hysteria .............. 491

Holberton's case of slow pulse...... 16

Homœopathy exposed ............ 282

Homœopathy and Dr. Ure.......... 561

Hospital Reports, Guy's .......... 225

Hospitals, London, deaths in the .... 318

Hudson on fever.................. 273

Hughes on abdominal effusion ...... 233

Hughes on malignant disease of lungs 239

Humerus, disarticulation of the .... 376

Humerus, reduction of dislocation of 403

Hydatids, case of ................ 291

Hydrocephaloid disease............ 20

Hydronephrosis .................. 219

Hydropathic establishment, Schmitz' 362

Hydropathy, Claridge on .......... 362

Hypochondriasis, Robarts on ...... 562

Hysteria, Hocken on.............. 491

Hysteria, pathology of ............ 493

Hysterical amaurosis.............. 492

I.

Iliac artery, ligature of the ........ 376

Importance of the microscope ...... 183

Inauguration of Broussais's statue .. 510

Indigestion, Dr. Dick on .......... 65

Indigestion, nitrate of silver in...... 65

Influence of the seasons on mortality 320

Inhalation, poisoning by .......... 569

Inoculation for pannus . .......... 264

Insane, religious delusions of the.... 423

Insanity, Leuret on ................ 399
Insanity, personal restraint in ...... 424
Institution for spinal diseases ...... 279
Intestino-vesical fistula ............ 249
Introduction of syphilis into Scotland 561
Ioduret of sulphur for porrigo ...... 268
Ioduret of sulphur for lepra & psoriasis 269
Ioduretted mercurial syrup for syphilis 202
Ischuria renalis ................... 55
Isle of France, hæmaturia in ...... 62

J.

Jackson on disease of the cerebellum 570
Jacksonian prize .................. 300
Jager's clinique ................... 263
Jamison on phrenology ............ 279
Jobert's case of ligature of the carotid 375
Johnson's case of hypertrophy of heart 276
Johnson, Athol, on paraplegia ...... 575
Joints, subcutaneous wounds of the.. 113
Jones on fracture of the cervix uteri 3

K.

Kidneys, malposition of the ........ 41
Kidneys, Rayer on diseases of the .. 51
Kidneys, Rayer on morbid formations 219
Kidneys, dropsy of the ............ 219
Kidneys, cysts of the .............. 220
Kidneys, worms in the ............ 221
Kidneys, foreign bodies in the ...... 223
Kidneys, mobility of the .......... 224
Kidneys, hypertrophy of the........ 267
Kidneys, Robinson on disease of the.. 447
Kidneys, granular disease of the .... 447
King on fracture of the patella...... 247

L.

Lane on the blood corpuscle ........ 248
Larrey, Baron, his career .......... 202
Lateral curvature of the spine ...... 109
Leamington .... ................... 87
Leamington, analysis of waters of .. 88
Leamington, saline springs of ...... 90
Leamington, sulphur springs of...... 91
Leamington, chalybeate spring at.... 92
Leucorrhœa ...................... 380
Leuret on insanity ................ 399
Leuret on the treatment of insanity.. 440
Ligature of the carotid artery ...... 244
Ligature of the carotid ............ 375
Ligature of the common iliac ...... 376
Lightning, death by .............. 316
Lithectasy ....................... 467
Lithectasy, description of .......... 589
Lithontriptics..................... 468
Lithotomy ........................ 485
Lithotomy, statistics of............ 486
Lithotrity ........................ 475
Lithotrity, statistics of ............ 477
Liver, malignant disease of the...... 260
Louis on typhoid fever ............ 515
Lunatic Asylum, Dundee Royal...... 545
Lungs, Addison on the anatomy of the 32
Lungs, expansion of the .......... 226
Lungs, exercise of the ............ 230
Lungs, malignant disease of the...... 239
Lungs, fungoid disease of the ...... 239
Lungs, gangrene of the............ 256

M.

Machardy on amputations.......... 172
Macleod on rheumatism............ 403
Malignant disease of the lungs...... 239
Malignant disease of the liver ...... 260
Malposition of the kidneys ........ 41
Mania, with and without paralysis .. 20
Marriage, statistics of ............ 307
Marsupial embryo, nourishment of the 143
Marsupial mamary fœtus .......... 146
Marsupial mamary organs.......... 148
Marsupial mamary pouch .......... 149
Marsupialia, gestation of the........ 141
Matlock ......................... 77
Medical reform, Crook on.......... 162
Medical studies, use of microscope in 165
Medical charities of Carlisle........ 277
Medical relief to the poor .......... 324
Medicinal properties of fish-liver oil.. 215
Medicine, memoirs of the Academy of 369
Medico-Chirurgical Transactions .... 1
Memoir of Dr. Birkbeck ............ 494
Memoirs of the Academy of Medicine 369
Menorrhagia ...................... 382
Menses, first appearance of the...... 377
Menses, influence of leucorrhœa on.. 380
Menstrual fluid, analysis of the...... 379
Menstruation, Boismont on ........ 377
Menstruation, influence of, on disease 382
Menstruation, influence of disease on 384
Microscope, Bennett on the ........ 165
Microscope, importance of the...... 183
Midwifery in China .............. 565
Miliary sweat .................... 370
Milky urine, cases of .............. 536
Mobility of the kidneys............ 222
Montgomery on cancer of the womb 568
Moral revulsion in insanity....... 399 440
Morbid formations in the kidneys .. 219
Mortality, statistics of ............ 305
Murders ......................... 315
Muscular rheumatism.............. 420
Museum of College of Surgeons .... 297

N.

Navy, health of the .............. 358
Nephritis......................... 52
Nephritis in parturient women ...... 54
Nephritis, chronic, treatment of .... 54
Nephritis, complications of ........ 55
Nephritis, gouty....... ..... .... 56
Nephritis, rheumatic. ............ 57
Nervous system, Hall on the........ 18
Nervous system, comparative anatomy 168
Nervous system, physiology of the .. 503
Neuralgia of the bladder . ........ 208
Neuralgic rheumatism ............ 422
New theory of tinea .............. 543

Nitrate of silver for indigestion .... 65
Nitrate of silver in gonorrhœa...... 281
Norwich, lithotomy at .... ...... 486
O.
Oil, fish-liver, properties of ........ 215
Old humoral pathology............ 183
Operation for stammering.......... 212
Operations of importance, Cooper on, 242
Operations for aneurysm .......... 243
Ophthalmic diseases in Vienna...... 264
Ophthalmology in Austria.......... 262
Orfila on poisoning by arsenic ...... 371
Orfila on hanging ................ 384
Organ, how to map out an ........ 564
Organized tissues, division of ...... 427
Organs of digestion, Dick on derangements of .................... 65
Os uteri, closure of the............ 573
P.
Paraplegia, Athol Johnson on ...... 575
Parturient women, treatment of .... 567
Parturition ...................... 565
Percussion, the art of.............. 562
Percussion, the gamut in .......... 564
Pericarditis and carditis............ 409
Pericarditis, symptoms of.......... 409
Periosteal rheumatism ............ 422
Personal restraint in insanity ...... 424
Pharmacy in France .............. 283
Pharmacy in England.............. 285
Phenomena of respiration .......... 458
Phlebitis ........ ............ 396
Phlegmasia dolens ................ 397
Phthisis terminating in hydrocephalus 292
Phthisis, &c. diuretics in .......... 559
Physiological obs. mode of conducting 507
Plague, recent works on the........ 538
Plague, contagiousness of .......... 538
Plague, symptoms of ...... ...... 539
Plague, pathological anatomy of ... 540
Plague, treatment of the .... ..... 541
Pleurisy, case of.................. 534
Poisoning by arsenic, Orfila on...... 371
Poisoning by arseniuretted hydrogen, 569
Poor-law medical relief ............ 324
Predisposing causes of consumption.. 542
Prolapsus ani, remedy for.......... 567
Pulmonary tissue, diseases of ...... 465
Pulmonary emphysema............ 465
Pus in the veins .................. 394
Q.
Quinine, hiccup cured with ........ 192
R.
Raciborski on the venous system .... 387
Rail-road travelling .............. 69
Rayer on diseases of the kidneys .... 51
Rayer on farcy .................. 160
Rayer on morbid formations in kidneys 219
Recent works on the plague ........ 538
Reduction of dislocation of the hip .. 271
Rees' case of abdominal effusion .... 233
Rees on the blood corpuscle ........ 248
Registrar-general, report of the ..... 305
Religious delusions of the insane .... 423
Remarkable cases, by Dr. Dickson .. 289
Remittent fevers.................. 369
Renal hæmorrhage................ 61
Renal fistulæ .................... 219
Report of the registrar-general ...... 305
Respiration, morbid sounds of ...... 459
Respiration, phenomena of ........ 458
Respiratory murmurs, analysis of the, 457
Results of amputation, Potter on the, 34
Rheumatic fever.................. 405
Rheumatic fever, treatment of. .... 406
Rheumatic ophthalmia ............ 418
Rheumatism, Macleod on .......... 403
Rheumatism, varieties of .......... 404
Rheumatism, capsular ............ 414
Rheumatism, capsular, effects of .... 416
Rheumatism, muscular ............ 420
Rheumatism, neuralgic ............ 422
Rheumatism, periosteal .......... 422
Richerand, memoir of ............ 528
Robarts on hypochondriasis ........ 562
Robinson on granular dis. of kidneys, 447
Rosas, clinique of ................ 265
Rubbing-sound in pericarditis ...... 259
S.
Salt, brandy and.................. 163
Sanguineous temperament.......... 518
Sanson, memoir of................ 531
Santerelli's section of the cornea.... 263
Sciatic nerve, dissection of tumor from 553
Scirrhus of the colon .............. 266
Scotland, introduction of syphilis into, 561
Scrofula, walnut leaf-tea for........ 192
Scurvy, treatment of.............. 358
Seasons, influence of the, on mortality 320
Section of muscles in amaurosis ..... 209
Section of muscles in blindness ..... 211
Serous exhalations, influence of air on 109
Setons, Marshall Hall on .......... 260
Setons, use of, in chronic inflam..... 261
Shaw's letter on M. Longet's work .. 507
Skin, warty excrescences on the .... 271
Slow pulse, Holberton's case of ..... 16
Sodon on displacem. of bicipital tendon 43
Sounds of the heart, Cruveilhier on.. 193
South American station............ 358
Spas of England, Granville's ........ 68
Spinal column, Hawkins on cancer of, 10
Spinal marrow in animals .......... 170
Spine, Guerin on lateral curvature of, 109
Spine, institution for diseases of the.. 279
Spittal's case of aneurism .......... 573
Spontaneous dislocations, Stanley on, 26
Stammering, operation for.......... 212
Stumps, mode of dressing .......... 38
Stanley on spontaneous dislocations.. 26
Stanley on tumors of the pelvis ..... 48
Statue of Broussais, inauguration of . 510

Steatomatous tumor in the back .... 246
Stomach-cough .................. 67
Stomach, ulceration of, from arsenic.. 232
Stomach, cancer of, Dr. Watson on .. 551
Stone in the bladder, Willis on...... 467
Stone, solution of ............... 468
Stone, Vichy waters for ........... 470
Studentships in anatomy ........... 299
Study in Vienna.................. 265
Strumous dyspepsia ............... 132
Subcutaneous surgery, Guerin on.... 109
Subcutaneous wounds ............. 112
Subcutaneous wounds of the joints .. 113
Suicide in France ................ 198
Suicide and cross-way burials ...... 281
Suicide, statistics of .............. 309
Sudden death, cases of ............ 316
Surgeons, payment of ............. 283
Surgeons, Royal College of ......... 295
Surgeons, Royal Coll. of, regulations, 296
Surgeon's, Royal College of, museum, 297
Surgery, Cyclopædia of............ 149
Surgery, ophthalmic, in Vienna .... 262
Surgery in Austria................ 262
Swan's comparative anatomy ....... 168
Synovial vessels.................. 430
Syphilis, ioduretted mercurial syrup for 202
Syphilis, introduction of, into Scotland 561
Sylvester on phlebitis.............. 8

T.

Taylor on poisoning with arsenic .... 231
Temperament, Andral on sanguineous, 518
Temperament, the lymphatic........ 519
Temperature, influence of, on mortality 321
Tender system .................. 336
Tetanus ........................ 20
Thigh, fungoid disease of the ....... 241
Thomson on tumors .............. 266
Tinea, new theory of.............. 543
Todd's Cyclopædia of Anatomy...... 140
Towns, diseases of................ 319
Toxicology in England ............ 231
Toynbee on diseases of the ear ...... 42
Toynbee on non-vasc. animal tissues, 426
Trachea, smallness of the .......... 228
Transactions of College of Surgeons.. 298
Transmission of glanders .......... 532
Traumatic gangrene, amputat. during, 552
Treacle and water for burns ........ 551
Tuberculous consump. Campbell on, 129
Tumor, dissect. of, from sciatic nerve, 550
Tumors, diagnosis of.............. 266
Tumors of thorax, painful nervous .. 554
Typhold fevers .................. 369
Typhoid fever, Louis on............ 515

U.

Ure on gouty concretions .......... 6

Ure and homœopathy.............. 561

Urine, milky, cases of.............. 536

Uterus, deficiency of the .......... 41

V.

Vaccination, Gregory on .......... 4
Vallance on brandy and salt ........ 163
Varicose veins, Velpeau's operation for 555
Varix ......................... 398
Vatican, prohibit. of animal mag. by, 213
Vegetable origin of tinea ........... 543
Veins, structure of................ 388
Veins, pus in the ................ 394
Veins, air in the.................. 395
Velpeau on abscess of iliac region .. 549
Venous system, history of the ...... 387
Venous system in the embryo ...... 390
Venous circulation................ 390
Venous absorption ............... 392
Venous system, pathology of the .... 393
Vesiculæ seminales, diseases of the .. 208
Vicarious menstruation............ 382
Vichy waters for stone ........... 470
Vienna, ophthalmic surgery in ...... 262
Vienna, plan of study in .......... 265
Violent deaths................... 307
Vitreous body, nourishment of the .. 437
Voice, physiology of the .......... 276

W.

Walnut-leaf tea for scrofula ........ 192

Walshe on endosteitis ............ 154

Warm water for colica pictonum .... 40

Warty excrescences on the skin .... 271

Water, purification of ............ 275

Wells on encephalocele............ 150

Wells on epistaxis .............. 156

Wilde on ophthalmic surgery ....... 262

Willis on stone in the bladder ...... 467

Wilson on colica pictonum......... 40

Wilson on aneurism of sup. mes. art. 45

Womb, Montgomery on cancer of the, 568

Worms in the kidneys ............ 221

Wunderlich on French physic ...... 177

Y.

Youth, Dr. Barlow on diseases of.... 225

### BIBLIOGRAPHICAL RECORD.

1. Pathology, founded on the Natural System of Anatomy and Physiology: a Philosophical Sketch, &c. &c. By ALEX. WALKER. Second Edition. 8vo. pp. 162. Churchill, London, 1841.

---

2. Researches into the Causes, Nature, and Treatment of the most prevalent Diseases of India, and of Warm Climates generally. By JAMES ANNESLEY, F.R.S. F.S.A. late President of the Medical Board, Madras. Second Edition, 8vo. pp. 607. Longman and Co. 1841.

☞ *This is a most valuable concentration of Mr. Annesley's large and magnificent work published a few years ago.*

---

3. The London and Edinburgh Monthly Journal of Medical Science. Conducted by J. R. CORMACK, M.D. Ed. Nos. X. and XI. for Oct. and Nov. 1841. London, Bailliere.

---

4. The Phrenological Journal and Magazine of Moral Science. Published quarterly. No. XVI. October 1, 1841. Price 2s. 6d.

---

5. Illustrations of the Comparative Anatomy of the Nervous System. By JOSEPH SWAN. Part VII. (last) price 7s. 4to. Oct. 1841. Longman and Co.

---

6. The Diseases of Children: their Symptoms and Treatment, &c. &c. By GEORGE AUGUSTUS REES, M.R.C.S. Surgeon to the General Dispensary for Children, &c. &c. Small Octavo, pp. 300, Highley, 1841.

---

7. Essais sur la Methode Sous-cutanée, contenant deux Memoirs sur les Plaies Sous-cutanées en general. Par le Docteur JULES GUERIN, Directeur de l'Institute Orthopedique de Paris. 1841.

---

8. Memoire sur l'Etiologie Generale des Deviations Laterales de l'Epine, par Retraction Musculaire Active, &c. Par le meme Auteur. 1841.

---

9. Memoire sur un Cas de Luxation Traumatique de la Seconde Vertebræ Cervicale, &c. Par le meme Auteur.

10. Recherches sur les Luxations Congenitales, &c. Par le meme Auteur.

---

11. Memoire sur l'Intervention de la Pression Atmospherique dans le Mechanisme des Exhalations Sereuses, &c. Par le meme Auteur. Paris, Aout. 1841.

---

12, Clinical Researches on Auscultation of the Respiratory Organs, and on the first Stage of Phthisis Pulmonalis. By JULES FOURNET, Intern of the Parisian Hospitals. Translated by THOMAS BRADY, M.B. Professor of Medical Jurisprudence in Dublin, Part I. price 7s. Churchill, London, Oct. 1841.

---

13. Principles of General and Comparative Physiology, intended as an Introduction to the Study of Human Physiology, &c. By WILLIAM B. CARPENTER, M.D. Lecturer on Physiology, Bristol. 8vo. pp. 578. Churchill, London, Oct. 1841.

---

14. A Treatise on the Nature, Causes, and Treatment of Erysipelas. By THOS. NUNNELEY, Lecturer in the Leeds School of Medicine, &c. 8vo. pp. 307. Churchill, London, 1841.

---

15. On the Remote Cause of Epidemic Diseases. By JOHN PARKIN, M.D. &c. 8vo. pp. 198. Hatchard and Son, London, Oct. 1841.

---

16. Guy's Hospital Reports. No. XIII. for October, 1841. Edited by Drs. BARLOW and BABINGTON. Highley, 1841.

---

17. A Treatise on the Oleum Jecoris Aselli, or Cod Liver Oil, as a therapeutic agent in certain forms of Gout, Rheumatism, and Scrofula. By JOHN HUGHES BENNETT, M.D. &c. 8vo. pp. 180. Highley, Oct. 1841.

---

18. Observations on the Analogy between Ophthalmic and other Diseases. Being the substance of a Paper read at King's College, London. By CHARLES VINES, M.R.C.S. 8vo. sewed, pp. 32. H. Renshaw, Strand, Oct. 1841.

19. The Prescriber's Pharmacopœia; containing all the Medicines in the London Pharmacopœia, arranged in classes according to their Action, with their Composition and Dose. By a Practising Physician. Duodecimo, pp. 118, price 2s. 6d. Churchill, London.

☞ *Never was half a crown better spent than in the purchase of this little " Thesaurus Medicaminum." This little work, with our visiting book and stethoscope are our daily companions in the carriage.*

---

20. A Manual of Electricity, Magnetism, and Meteorology. By DIONYSIUS LARDNER, D.C.L. F.R.S. &c. (Cabinet Cyclopædia,) Vol. 1. pp. 440. Longman and Co. 1841.

---

21. The Naturalist's Library. Icthyology, Vol III. W. Lizars, 1841.

---

22. Observations on Tuberculous Consumption, containing new views on the Nature, Pathology, and Cure of that Disease, &c. By D. S. CAMPBELL, M.D. Senior Physician to the St. Marylebone General Dispensary. 8vo. pp. 404, with plates. Bailliere, 1841.

---

23. Pharmaceutical Transactions. Edited by JACOB BELL. No. 5, for November, 1841.—Also No. 4, for October.

---

24. On the present State of the Medical Profession in England. By ROBERT E. GRANT, M.D. Octavo pp. 98. Bailliere, 1841.

---

25. The Cyclopædia of Anatomy and Physiology. By B. TODD, M.D. &c. Part 22. Sherwood and Co. 1841.

---

26. Cyclopædia of Practical Surgery, &c. Part X. Edited by W. B. COSTELLO, M.D. Sherwood and Co. Nov. 1841.

---

27. The Dublin Journal of Medical Science, &c. No. 59. Nov. 1841.

☞ *In Exchange*

---

28. Dissertationis de Ulcere Ventriculi Perforante Particula Prior, &c. Auctor EDUARDUS AUGUSTUS DAHLERUP, M.D. Havniæ, 1840.

☞ *A very creditable thesis. Eighteen cases of this dreadful disease are detailed, and many of our countrymen are quoted.*

---

29. Quid faciant Ætas, annique tempus ad frequentiam et ducturnitatem Morborum hominis Adulti? Disquisitio Medico-Statistica. Autore E. FENGER, L.M. Univ. Havn. 1841.

---

30. Memoires de l'Academie de Medicine. Tome Neuvieme. Octobre, 1841. Quarto, Bailliere, Paris et Londres.

---

31. The Principles of Physiology applied to the Preservation of Health, and to the Improvement of Physical and Mental Education. By A. COMBE, M.D. Tenth Edition, with 13 wood-cuts. Maclachlan and Co. Edinb. Simpkin and Marshall, London. Aug. 1841.

☞ *This edition is carefully revised, and considerably enlarged.*

---

32. England and her Colonies, considered in relation to the Aborigines; with a proposal for affording them relief. Published by the Aboriginal Protection Society.

☞ *An eloquent Address in favour of several millions of the coloured race in our Transatlantic, Australian, Polyhesian, African, and Asiatic possessions.*

---

33. A Manual of General Therapeutics; with Rules for prescribing, and a copious collection of Formula. By D. SPILLAN, M.D. A.M. &c. 8vo. pp. 458, very small type, and copious Index.

☞ *This will prove a great favourite with the active practitioner, who has not time to wade through elaborate works on therapeutics. It is an excellent compilation, some portions of which we shall notice in our next Number.*

---

34. Elements of Chemistry, including the most recent Discoveries and Applications of the Science to Medicine, Pharmacy, and the Arts. By ROBERT KANE, M.D. M.R.S.A. Professor of Natural Philosophy to the Royal Dublin Society, &c. Part 3, with Contents and Index, completing the work. Dublin, Hodges and Smith. London, Simpkin and Co. 1841.

---

35. Interesting Facts connected with the Animal Kingdom; with some remarks on the Unity of our Species. By J. C. HALL, M.D. 8vo. pp. 301. Whittaker, London, Nov. 1841.

---

36. Memoire sur la Revulsion Moral dans le Traitement de la Folie. Par F. FLEURET, Medecin en Chef à l'Hospice de Bicetre, &c.

37. Anatomie Pathologique du Corps Humain, &c. Par J. CRUVELHIER. 38th Fasciculus. Bailliere, Nov. 1841.

38. Philosophic Nuts; or the Philosophy of Things, as developed from the Study of the Philosophy of Words. By EDWARD JOHNSON, Esq. No. 10, for October, 1841. Simpkin and Co.

*** *The more we have read of Mr. Johnson's "Philosophy of Things" the more we are convinced that nothing equal to it in deep thought and laborious research has appeared in the English language since the days of Locke and Horne Tooke.*

39. Elements of Physiology. By J. MÜLLER, M.D. Translated from the German, with Notes, by WILLIAM BALY, M.D. &c. Illustrated with Steel Plates and numerous Wood-cuts. Part VI. containing Mind, Generation and Development. London, Taylor and Walton, price 10s. 6d.

40. Practical Astronomy for the Unlearned. With numerous Engravings. By the Rev. GEORGE JEANS, of Pembroke College, Oxford. 8vo. pp. 288. Capes & Co. London, 1841.

*** *To those who wish to acquire a light but lucid knowledge of the delightful science of Astronomy, this little volume will prove a great acquisition.*

41. Physiology for the Public, &c. No. 1, November, and No. 2, December, 1841. Price 1s. each. In a series of Lectures. By G. T. HAYDEN, A.B. M.R. &c. Orr, London. Fannin and Co. Dublin.

*** *Mentioned in the Bib. Notices.*

42. An Investigation into the proposed Scheme of Medical Reform, in reference to Chemists and Druggists, &c. By G. CROOK, M.P.S. 8vo. price 1s. Hastings, Carey-street, London, Nov. 1841.

43. A History of British Birds. By WILLIAM YARRELL, F.L.S. Illustrated by wood-cuts of each species, and numerous vignettes. Parts 26, 27, and 28, price 2s. 6d. each. Van Voorst, London, Nov. 1841.

44. The Maryland Medical and Surgical Journal, and Official Organ of the Medical Department of the Army and Navy of the United States. Published under the auspices of the Medical and Chirurgical Faculty of Maryland. No. 1 of Vol. 2. July, 1841.

*** *We would be happy to exchange Journals, but we do not know through what channels as yet.*

45. On the Employment of the Microscope in Medical Studies. A Lecture, introductory to a Course of Histrology. By J. HUGHES BENNETT, M.D. Lecturer on Clinical Medicine, &c. Ed. 1841.

46. An Address to Parents and Guardians relative to the Treatment of Imbecile Children.

*** *This is a sensible address, and we believe the lady who professes to undertake this responsibility is very talented and respectable. Information may be received at* 33, *Great Portland Street.*

47. The Anatomy of the Arteries of the Human Body, with its Application to Pathology and Operative Surgery. In Lithographic Drawings, with Practical Commentaries. By RICHARD QUAIN, Professor of Anatomy, &c. in University College. The Delineations by JOSEPH MACLISE, Esq. Surgeon. Part 10, price 12s. Taylor and Walton, Dec. 1841.

48. The Naturalist's Library. Entomology. Vol. VIII. Edinburgh, Lizars. London, Highley, 1841.

49. The Double Flap and Circular Amputations Contrasted. Being an Abstract of a first Prize Essay at the University of Edinburgh. By F. N. MACHARDY, A.M. and M.D. Surgeon. London, Simpkin and Marshall, Dec. 1841.

50. The Chemist; a Reporter of Chemical Discoveries and Improvements, &c. &c. Edited by CHARLES WATT, Esq. Lecturer on Chemistry, and JOHN WATT, Jun. Vol. 2, 1841.

*** *This is a most meritorious Journal, (published monthly by R. Hastings, Carey-street) which we regret to have left so long unnoticed. In our next and succeeding Numbers we shall take care to pay attention to the work.*

